# Apelinergic System Affects Electrocardiographic Abnormalities Induced by Doxorubicin

**DOI:** 10.3390/biomedicines13010094

**Published:** 2025-01-03

**Authors:** Kasper Buczma, Hubert Borzuta, Katarzyna Kamińska, Dorota Sztechman, Katarzyna Matusik, Jan Pawlonka, Michał Kowara, Barbara Buchalska, Agnieszka Cudnoch-Jędrzejewska

**Affiliations:** Chair and Department of Experimental and Clinical Physiology, Laboratory of Centre for Preclinical Research, Medical University of Warsaw, Banacha 1b, 02-097 Warsaw, Poland; kasper.buczma@wum.edu.pl (K.B.); hubert.borzuta@student.wum.edu.pl (H.B.); kkaminska@wum.edu.pl (K.K.); dorota.sztechman@wum.edu.pl (D.S.); katarzyna.matusik@wum.edu.pl (K.M.); jan.pawlonka@wum.edu.pl (J.P.); michal.kowara@wum.edu.pl (M.K.); buchalskabarbara@gmail.com (B.B.)

**Keywords:** apelinergic system, cardiotoxicity, doxorubicin, electrocardiography, transthoracic echocardiography

## Abstract

**Background/Objectives**: Anthracyclines remain a pivotal element of numerous tumor management regimens; however, their utilization is associated with a range of adverse effects, the most significant of which is cardiotoxicity. Research is constantly being conducted to identify substances that could be incorporated into ongoing cancer chemotherapy to mitigate anthracycline-induced cardiotoxicity. Recently, the apelinergic system has received a lot of attention in this field due to its involvement in cardiovascular regulation. Therefore, the aim of our study was to investigate the ability of the apelinergic system to inhibit the cardiotoxic effects of anthracycline—doxorubicin (DOX). **Methods**: In this study, 54 Sprague–Dawley rats were divided into seven groups and received intraperitoneal injections with DOX once a week for 4 consecutive weeks. The osmotic pumps provided a continuous release of NaCl (control groups), apelin-13 and elabela at two different doses, and the apelin receptor (APJ) antagonist ML221. Electrocardiography (ECG) and transthoracic echocardiography (TTE) with assessment of left ventricular (LV) systolic parameters were conducted on the first and last days of the experiment. **Results**: Lower doses of APJ agonists prevented the prolongation of QT and QTc intervals induced by DOX, while higher doses of these drugs exerted no such effect. The TTE examination confirmed DOX-induced LV systolic dysfunction. Moreover, the TTE examination revealed an improvement in the LV systolic parameters in the DOX-treated groups that were simultaneously administered APJ agonists. **Conclusions**: Our findings support the use of apelin and elabela as potential cardioprotective agents against anthracycline-induced cardiotoxicity.

## 1. Introduction

Doxorubicin (DOX) is a highly effective anti-neoplastic agent included in the group of drugs known as anthracyclines used to treat several types of cancer, such as breast cancer, leukemia, lymphomas, sarcomas, and many others. It exerts its action mostly due to inhibition of topoisomerase-II and generation of free radicals, both leading to subsequent DNA damage. The most serious adverse effect of this drug is cardiotoxicity, which limits its clinical use [1]. The risk of developing cardiotoxicity increases with the cumulative dosage [2,3]. Doxorubicin-induced cardiotoxicity (DIC) is characterized by a broad spectrum of symptoms ranging from minor electrocardiographic abnormalities, arrhythmias, left ventricular dysfunction to pericarditis and cardiomyopathy [4].

Various diagnostic methods are used to detect cardiac dysfunction induced by DOX treatment. The current European Society of Cardiology (ESC) Guidelines on cardio-oncology recommend cardiovascular toxicity risk stratification before anticancer therapy and monitoring of cardiovascular complications during cancer therapy using electrocardiography (ECG), cardiac serum biomarkers (cardiac troponin and natriuretic peptides), and cardiac imaging (transthoracic echocardiography or cardiac magnetic resonance imaging) [5].

In preclinical research of cardiotoxicity following chemotherapeutic agents, one component of myocardial health assessment is the evaluation of electromechanical function using ECG assessment. In these studies, great attention is focused on the presence and frequency of supraventricular as well as ventricular arrhythmias and the assessment of the morphology of the waveforms, their amplitudes and lengths, and the morphology and lengths of the individual segments and intervals.

According to Jensen and co-workers (1984), the most consistent ECG changes in rats (strain not specified) observed with DOX treatment (with the cumulative dose higher than 10 mg/kg) were a reversible prolongation of the QRS complex and a progressive lengthening of the QT interval, while changes in the R-, S-, and T-wave voltages were more variable. What is interesting is that the ECG toxicity was more pronounced when the DOX was administrated in a weekly interval [6].

Villani and co-workers (1986) evaluated the dose- and time-dependence of the effect of DOX on ECG to establish the relationship between structural alterations of the myocardium and ECG changes in rats (female Sprague–Dawley, weighing 120–130 g). The group administrated the DOX at a dose of 1.5 or 3.0 mg/kg, three times for 3 days. They found a dose-dependent, but reversible prolongation of the QRS complex, and a dose-dependent and progressive irreversible increase in interval between the beginning of the QRS complex and the apex of the T-wave (QaT) and in interval between the beginning of the S-wave and the apex of the T-wave (SaT) duration [7].

Interestingly, gender differences were demonstrated in DOX-treated animals by another group. The results of Warhol et al. (2021) showed that DOX administration induced significant ECG modulation in female C57BL/6 mice alone. In addition, it was shown that DOX treatment (20 mg/kg) induced a shortening of P-wave and QRS duration, while DOX treatment (30 mg/kg) induced a prolongation of QTc and RR interval in anesthetized but not in conscious female mice. These data suggest significant gender differences and anesthesia-induced differences in ECG response to DOX. Moreover, this research group showed P-wave and QTc shortening as well as PR and RR interval prolongation in anesthetized versus conscious saline-treated mice [8].

Mentioned effects have also been observed in a study on patients undergoing DOX therapy. For example, QT interval prolongation is observed in up to 30% of patients treated with DOX [9]. Kinoshita et al. (2021) investigated the long-term visual fluctuations in the ECG waveforms in patients suffering from DOX-induced chemotherapy-related cardiac dysfunction (CTRCD) to identify ECG indices for the early detection of this cardiotoxicity [10]. The CTRCD was detected at a median of 475 days after initiating chemotherapy. ECG indicators preceding the development of CTRCD were evaluated 93 days before the detection of CTRCD. At the onset of CTRCD, the most significant ECG change was flattening of the T-wave in the V3–V6 leads (12 patients, 80%). In addition, QaT interval extension was observed in leads I and aVL—over the lateral heart wall (10 patients, 66%), leads II, III, and aVF—over the inferior heart wall (60%) and leads V3–V6 over the anterior heart wall (10 patients, 73%). These ECG alterations were not seen before medical treatment but were during CTRCD development and progression [10]. In a number of cardiovascular diseases, QT interval prolongation is associated with the presence of myocardial fibrosis, as observed on cardiac magnetic resonance (CMR) [11].

Despite advances in our understanding of the pathogenesis, modification of chemotherapy regimens, and earlier diagnosis, the risk of developing anthracycline-induced cardiotoxicity and subsequent clinical heart failure decompensation remains at approximately 2–4% [12].

Currently, the only FDA-approved cardioprotective agent against anthracyclines is dexrazoxane. Its mechanism of action includes inhibition of apoptosis and necroptosis, as well as mitigation of chemotherapy-induced inflammation [13]. The use of dexrazoxane in children treated with high doses of doxorubicin for sarcoma attenuated myocardial remodeling and deterioration of systolic function. It should be noted that this effect may be gender specific, with a greater impact in females [14,15]. Based on the available literature, the International Late Effects of Childhood Cancer Guideline Harmonization Group recommends the use of dexrazoxane in children when the cumulative doxorubicin or equivalent dose is at least 250 mg/m^2^ (moderate recommendation). In these cases, benefits of dexrazoxane therapy exceed potential adverse effects [16]. Another group of drugs extensively studied in the prevention of DIC are statins. There are conflicting data regarding the effects of statins as cardio-protectants. A meta-analysis of seven studies comprising a total of 2511 patients demonstrated that those prescribed with statins exhibited a reduced risk of developing cardiotoxicity compared to those not receiving statins. However, statins did not prevent the chemotherapy-related decline in the left ventricular ejection fraction (LVEF) [17].

Recently, the apelinergic system has received a lot of attention due to its involvement in cardiovascular regulation. The key molecule in this system is apelin—a group of peptides produced by C-terminal cleavage of the 77-amino acid precursor preproapelin [18]. Apelin (APLN) is produced by adipose tissue as well as the brain, heart, blood vessels, and kidneys. APLN has its own receptor—APJ—that is present in the heart, blood vessels, brain, lungs, kidneys, and adipose tissue [18,19]. Activation of the APJ by APLN initiates a phosphatidylinositol kinase/protein kinase B (PI3K/AKT), ERK1/2, and PLN-mediated signaling pathways [20]. Elabela—Apela/Toddler (ELA) is a new peptide classified as an apelinergic system component. The ELA amino acid sequence is at 25%, similar to the apelin, and therefore, it seems that ELA can activate the APJ and exert an apelin-like effect. ELA and apelin are differentially processed and hence their potency and half-lives might be different which could affect their in vivo effects [21]. The apelinergic system has been linked with several pathological conditions including chronic heart failure, diabetes, and obesity [22]. Dysregulation of the apelinergic system has been studied in animal models of heart failure (HF) and myocardial infarction, as reported in our previous research [23,24]. Downregulation of the apelinergic system in animal models has been shown to decrease exercise capacity under physiological stress, induce progressive HF, and susceptibility to cardiac oxidative stress [25].

According to our knowledge, there were only few studies on the influence of the apelinergic system on ECG conduction abnormalities in a different model of cardiac failure. In a rat (female Wistar albino rats, weighing 190–250 g) model of diclofenac sodium-induced cardiotoxicity, it was observed that decreased serum APLN concentration is associated with a higher heart rate (HR), prolongation of the QT interval, and QRS complex duration [26]. In another in vivo study, performed on the male C57Bl/6 mice, evaluating the effect of APJ stimulation on hemodynamic parameters, it was discovered that intraperitoneal injections of APLN resulted in an increase in HR [27]. Consistent results were obtained by Akhondali et al. who assessed the effect of co-administration of APLN and thyroxine (T4) on the mechanical and electrical activity of the heart in hypothyroid male Wistar rats (170–235 g). Both APLN monotherapy and in combination with T4 caused an increase in HR and QRS voltage [28]. There is also observation that APLN might shorten the pathologically prolonged QT interval due to the increasing inward rectifier potassium current (IK1) via PI3kinase-dependent signaling pathways in the mice (strain not specified) model [29].

Currently, there are no studies establishing the influence of the apelinergic system on doxorubicin-induced cardiac electrophysiology dysfunction. Thus, the aim of the present study was to investigate if the apelinergic system affects electrocardiographic abnormalities in the rat model of chronic DOX administration.

## 2. Materials and Methods

### 2.1. Animals

This study was performed on 54, adult male Sprague–Dawley rats weighted 350–400 g. All rats came from the Central Laboratory of Experimental Animals at the Center for Preclinical Research and Technology of the Warsaw Medical University. The experiments were carried out in accordance with the European Union guidelines for the care and use of laboratory animals (Council Directive 86/609/EEC of 24 November 1986) and were approved by the Second Local Ethical Committee for Animal Experiments in Warsaw (Resolution No. WAW2/087/2021 from 2 July 2021). The animals were housed in a mechanically ventilated room with controlled light day (12 h/12 h), controlled temperature (20–24 °C), and humidity of 55% (±10%). Rats were kept in standard plastic cages with metal lids of four individuals each, with free access to drinking water and standard feed for laboratory animals. All cages contained environmental enrichments in the form of plastic red, transparent to the rats, houses and paper tubes.

Only males were intentionally used in this study since the literature reports in recent years have shown that female hormones cause activation of the apelinergic system.

### 2.2. Experimental Protocol

**Figure 1:**
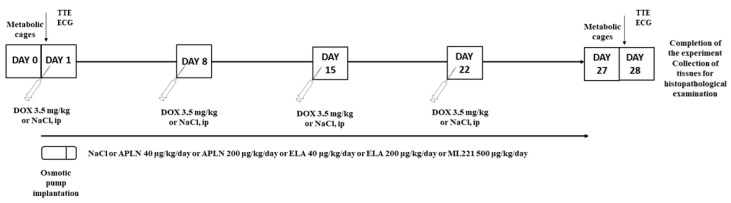
A schematic visualization of the experiment.

The procedure of the experiment is schematically presented in Figure 1. On the day “0”, animals were moved to metabolic cages for 24 h. On first day of the experiment, the animals were subjected to ECG and TTE under general anesthesia induced with intraperitoneal (ip) injection of ketamine (75 mg/kg of b.w.) with xylazine (7 mg/kg of b.w.). Subsequently, individuals in the control group received an ip injection of 0.9% sodium chloride, while those in the experimental groups received an ip injection of doxorubicin hydrochloride (dose 3.5 mg/kg of b.w.). The injections were repeated at weekly intervals, up to a total of four injections. All rats included in the experiment underwent surgical implantation of an osmotic pump (Alzet Corp., Cupertino, CA, USA), which was placed subcutaneously in the interscapular region. The rats received infusions of saline, apelin (dose 40 μg/kg of b.w./day or 200 μg/kg of b.w./day), ELA (dose 40 μg/kg of b.w./day or 200 μg/kg of b.w./day), or the APJ antagonist ML 221 (dose 500 μg/kg of b.w./day) with infusion rate of 2.5 μL/h for 28 days. One week after the last injection, the animals were subjected to repeat ECG and TTE under general anesthesia, and then sacrificed for tissue collection for histopathological evaluation.

Data collected from animals were excluded if the response to the administration of anesthetics differed significantly from that of other animals (too long a time to fall asleep/awake), or if the evaluation of osmotic pumps could indicate their potential malfunction.

The experimental groups *:

1. Control group (Control)—rats receiving saline i.p. (0.9% NaCl) in adequate volume every 7 days for 4 consecutive weeks (4 doses in total), and saline (0.9% NaCl) 2.5 μL/h in osmotic pump for 28 days starting on day 1.
2. Doxorubicin group (DOX)—rats receiving i.p. doxorubicin at the dose of 3.5 mg/kg/b.w. every 7 days for 4 consecutive weeks (4 doses in total), and saline (0.9% NaCl) 2.5 μL/h in osmotic pump for 28 days starting on day 1.
3. Apelin 40 group (APLN 40)—rats receiving i.p. doxorubicin at the dose of 3.5 mg/kg/b.w. every 7 days for 4 consecutive weeks (4 doses in total), and apelin-13 at the dose of 40 μg/kg b.w./day (2.5 μL/h) in osmotic pump for 28 days starting on day 1.
4. Apelin 200 group (APLN 200)—rats receiving i.p. doxorubicin at the dose of 3.5 mg/kg/b.w. every 7 days for 4 consecutive weeks (4 doses in total), and apelin-13 at the dose of 200 μg/kg b.w./day (2.5 μL/h) in osmotic pump for 28 days starting on day 1.
5. Elabela 40 group (ELA 40)—rats receiving i.p. doxorubicin 3.5 mg/kg/b.w. every 7 days for 4 consecutive weeks (4 doses in total) with Elabela 40 μg/kg b.w./day (2.5 μL/h) in osmotic pump for 28 days starting on day 1.
6. Elabela 200 group (ELA 200)—rats receiving i.p. doxorubicin 3.5 mg/kg/b.w. every 7 days for 4 consecutive weeks (4 doses in total) with Elabela 200 μg/kg b.w./day (2.5 μL/h) in osmotic pump for 28 days starting on day 1.
7. ML221 group (ML221)—rats receiving i.p. doxorubicin 3.5 mg/kg/b.w. every 7 days for 4 consecutive weeks (4 doses in total) with ML 221,500 μg/kg b.w./day (2.5 μL/h) in osmotic pump for 28 days starting on day 1.

* In the continuation of this article, abbreviated group names will be used for better readability.

### 2.3. Drugs and Reagents

The animals received ip injection of doxorubicin hydrochloride (MedChemExpress). The drug in powder form was suspended in distilled water in the ratio 10 mg of doxorubicin hydrochloride per 1 mL of distilled water. The measured volume of the solution thus prepared was then topped up with 0.9% sodium chloride to a total volume of 1 mL. According to the methodology for ip injection in rodents described by Morton et al. (2001) and Coria-Avila et al. (2007), rats were held in a supine position during injection, with the head positioned below the level of the abdominal cavity, and injections were administered in the region of the right iliac fossa to reduce the risk of adverse events in the form of enteral or intravesical administration of the drug or damage to the continuity of the large intestine [30,31]. Apelin-13, Elabela, and ML221 (4-oxo-6-((pyrimidin-2-ylthio) methyl)-4H-pyran-3-yl 4-nitrobenzoate) were purchased from MedChemExpress LLC (Sollentuna, Sweden). The ketamine hydrochloride and xylazine hydrochloride came from Centrowet (Białystok, Poland). The manufacturer of the 2 mL used osmotic pumps (ALZET Osmotic Pumps) recommends filling the pump to 90% of its capacity, i.e., 1.8 mL. The pumps were filled with a mixture of distilled water and NaCl to volume of 1.8 mL. Depending on the test group, either Apelin/Elabela 40 at a dose of 0.04 mg/average animal weight per group/day or Apelin/Elabela 200 or ML221 at a dose of 0.2 mg/average animal weight per group/day was added. The doses of 5 test groups were recalculated to ensure the release of the test substances over the 28-day duration of the experiment.

The doses were selected on the basis of our research on the apelinergic system and in vitro studies findings.

### 2.4. Electrocardiographic Examination

Electrocardiography was performed in all animals on the first and last day of the experiment. The animals remained under general anesthesia during the examination. The eyes of the animals were protected with gel to prevent dryness. The rats were laid on their backs and their paws were moistened with 0.9% sodium chloride to ensure optimal conductivity between the animals’ skin and the electrodes. The limb lead electrodes were connected to the animals’ paws using metal clamps. The examination was performed using a BTL-08 electrocardiograph (BTL Poland Sp. o.o., Warsaw, Poland). ECG recordings were recorded using CardioPoint software EKG C600 (BTL Poland). Two ECG recordings lasting 10 s each were obtained for each individual.

Measurements were made on the obtained recordings using the aforementioned software, according to the methodology for evaluating electrocardiographic recordings in rats described by Konopelski et al. (2016) [32]. The recording of the bipolar II limbic lead was analyzed.

The following parameters were evaluated:

- QRS complex

Its length indicates how long depolarization takes to spread across the ventricles. Wide QRS complexes are indicative of intraventricular conduction disturbances usually occurring during left or right bundle-branch blocks, cardiac ischemia, and heart failure [32].

- QT and QTc

The QT interval is the segment between the start of the Q-wave to the end of the T-wave. In rats, due to the difficulty in recording the Q-wave, the onset is taken as the beginning of the RS band. It describes the duration of depolarization and repolarization of the ventricular myocardium. Prolongation of QT indicates damage to the ventricular myocardium by toxins or myocardial disease [32]. QT prolongation is thought to be a useful exponent of drug-induced cardiotoxicity, as demonstrated in a number of studies [33,34,35,36].

It is commonly known that HR affects how long a person’s QT interval is. Generally speaking, as the ratio of systole to diastole lengths increases, an increase in HR shortens QT. As a result, a more objective measure of the depolarization and repolarization of ventricles is frequently a corrected QT interval (QTc), which accounts for variations in heart rate [32]. Due to the significantly higher HR in rats, in order to correctly calculate QTc, we used an adapted Bazett formula, previously used in the literature developed by Kmecova and Klimas:

$$\mathrm{QTc}=\frac{\mathrm{QT}}{\sqrt{\frac{\mathrm{RR}}{150\;\mathrm{ms}}}}$$

where QTc—corrected QT interval duration, QT—QT interval duration, RR—RR interval duration [37].

- HR

The number of heart contractions during a predetermined amount of time, generally one minute (beats per minute, or bpm), is represented by the heart rate (HR). In rats, the r–r interval—the segment from the peak of the R-wave to the peak of the next R-wave—is used for assessment. This feature is essential for correct calculation of the valid QTc value [32].

### 2.5. Transthoracic Echocardiographic (TTE) Examination

On the first and last days of the experiment, the TTE was performed with the use of Vivid i (G.E. HealthCare Technologies Inc.; Chicago, IL, USA) equipped with 11 MHz cardiac phase array probe (10S-RS; G.E. HealthCare Technologies Inc.). Echocardiography was performed on rats anesthetized with a combination of Ketamine (75 mg/kg b.w.; Vetoquinol, Gorzow Wielkopolski, Poland) and Xylazine (7 mg/kg b.w.; Vetoquinol, Gorzow Wielkopolski, Poland) given intraperitoneally (i.p.). After anesthesia induction, the thorax of each rat was covered with shaving cream and shaved. For the echocardiographic examination, the rats were placed on a heating plate in the left decubitus position. The protocol of echocardiographic examination was performed following the guidelines on TTE measurements in laboratory animals [38,39]. The analysis included the following measurements: ejection fraction (EF), fractional shortening (FS), heart rate (HR), stroke volume (SV) and cardiac output (CO), velocity time integral (VTI) and aortic diameter (Ao). Fractional shortening (FS) and ejection fraction (EF) were assessed from 2D-guided M-mode imaging in the parasternal short axis (SAX) view. Aortic diameter was measured by B-mode in the parasternal long axis (PLAX) view. The left ventricular outflow tract velocity time integral (LVOT-VTI) was recorded with pulsed-wave Doppler from apical 5-chamber view (A5C). SV was calculated with the use of VTI measurement multiplied by Ao. CO was calculated by multiplying SV by HR. CO was indexed to the body surface area (BSA).

### 2.6. Multimedia

The graphical abstract was created with BioRender.com. (accessed on 13 December 2024).

## 3. Results

### 3.1. Electrocardiographic Measurements

**Figure 2:**
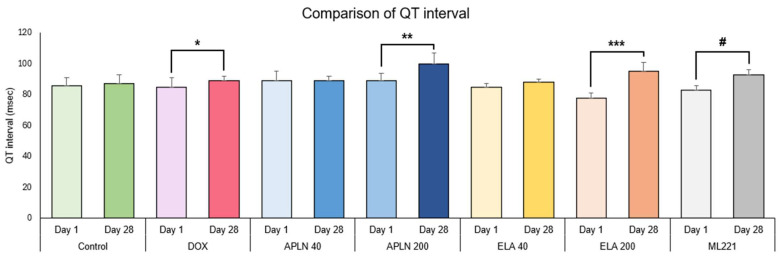
Comparison of QT interval between day 1 and day 28 within the groups. * *p* < 0.004 in DOX, ** *p* < 0.01 in APLN 200, *** *p* < 0.0001 in ELA 200, # *p* < 0.004 in ML221.

**Figure 3:**
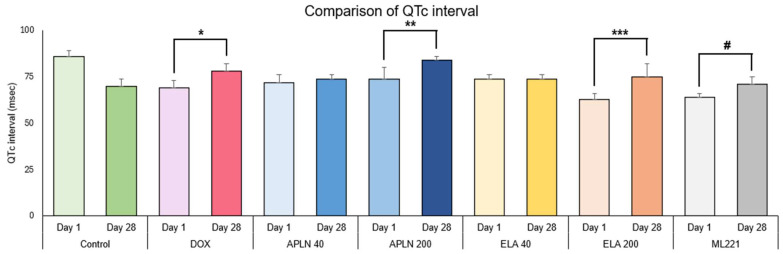
Comparison of QTc interval between day 1 and day 28 within the groups. * *p* < 0.005 in DOX, ** *p* < 0.01 in APLN 200, *** *p* < 0.001 in ELA 200, # *p* < 0.006 in ML221.

**Figure 4:**
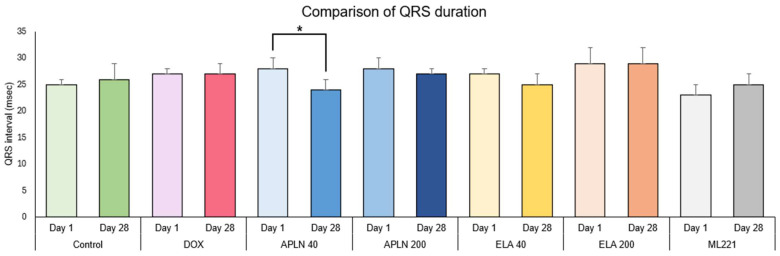
Comparison of QRS duration between day 1 and day 28 within the groups. * *p* < 0.0004 in APLN 40.

**Figure 5:**
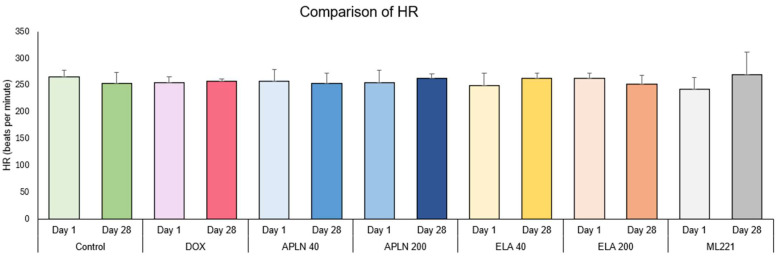
Comparison of HR between day 1 and day 28 within the groups.

In Figure 2, Figure 3, Figure 4 and Figure 5, the results are shown as comparisons between the values from the first measurement described as day 1 and the results from the second measurement described as day 28 within all groups.

#### 3.1.1. Comparison of QT Interval Between Day 1 and Day 28 in the Groups

One-way ANOVA followed with post hoc Duncan test showed no statistical significance in the Control group between measurements taken on days 1 and 28. The significant effect was observed in the DOX group (F 1.9 = 14.3, *p* < 0.004), * *p* < 0.004 (post hoc Duncan test), APLN 200 treatment (F = 1.9 = 8.9, *p* < 0.01), ** *p* < 0.01 (post hoc Duncan test), ELA 200 treatment (F 1.12 = 41.7, *p* < 0.0001), *** *p* < 0.0001 (post hoc Duncan test), and ML221 treatment (F 1.9 = 29.6, *p* < 0.005), # *p* < 0.004 (post hoc Duncan test). There was no statistical significance for the APLN 40 and ELA 40 treatments (Figure 2).

#### 3.1.2. Comparison of QTc Interval Between Day 1 and Day 28 in the Groups

One-way ANOVA followed with post hoc Duncan test showed no statistical significance in the Control group between measurements taken on days 1 and 28. The significant effect was observed in the DOX group (F 1.10 = 12.7, *p* < 0.005), * *p* < 0.005 (post hoc Duncan test), APLN 200 treatment (F 1.8 = 11.2, *p* < 0.01), ** *p* < 0.01 (post hoc Duncan test), ELA 200 treatment (F 1.12 = 18, *p* < 0.001), *** *p* < 0.001 (post hoc Duncan test) and ML221 treatment (F 1.10 = 11.6 *p* < 0.006), # *p* < 0.006 (post hoc Duncan test). There was no statistical significance for the APLN 40 and ELA 40 treatments (Figure 3).

#### 3.1.3. Comparison of QRS Duration Between Day 1 and Day 28 in the Groups

There was no statistical significance in the Control, DOX, APLN 200, ELA 40, ELA 200, and ML221 treatments. One-way ANOVA showed the significant effect of the APLN 40 treatment measured at day 1 vs. day 28 (F 1.17 = 20.9, *p* < 0.0002), * *p* < 0.0004 (post hoc Duncan test) (Figure 4).

#### 3.1.4. Comparison of HR Between Day 1 and Day 28 in the Groups

One-way ANOVA followed with post hoc Duncan test showed no statistical significance in the Control, DOX, APLN 40, APLN 200 treatments between measurements taken on days 1 and 28. There was no statistical significance for the ELA 40, ELA 200, and ML221 treatments (Figure 5).

### 3.2. Echocardiographic Measurements

**Figure 6:**
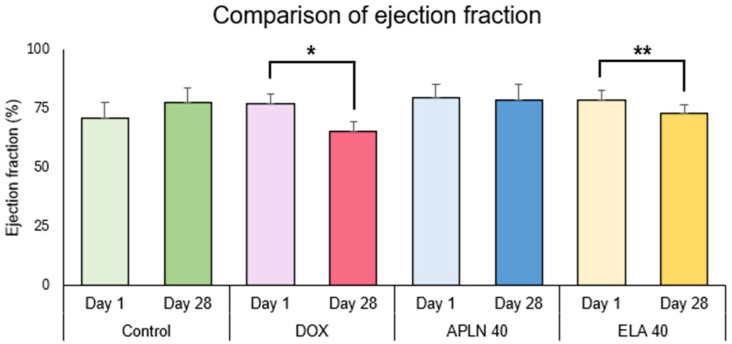
Comparison of ejection fraction between day 1 and day 28 within the groups. * *p* < 0.0003 in DOX, ** *p* < 0.01 in ELA 40.

**Figure 7:**
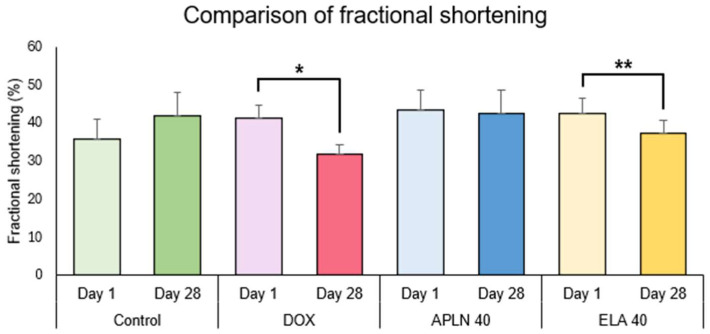
Comparison of fractional shortening between day 1 and day 28 within the groups. * *p* < 0.0002 in DOX, ** *p* < 0.01 in ELA 40.

**Figure 8:**
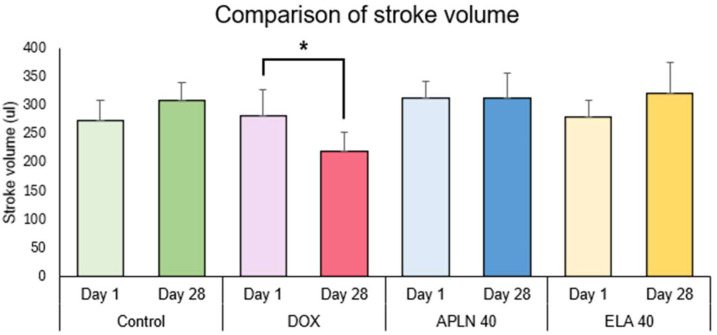
Comparison of stroke volume between day 1 and day 28 within the groups. * *p* < 0.01 in DOX.

**Figure 9:**
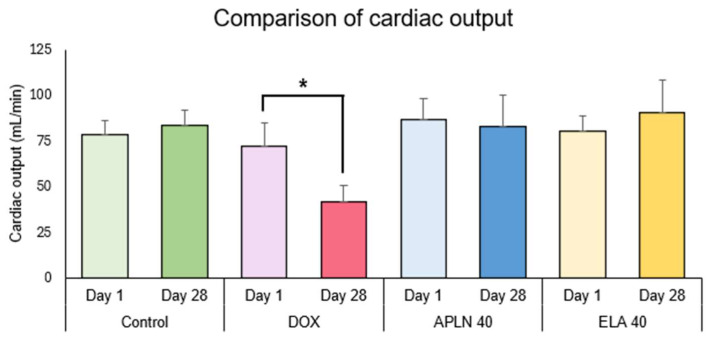
Comparison of cardiac output between day 1 and day 28 within the groups. * *p* < 0.003 in DOX.

In Figure 6, Figure 7, Figure 8 and Figure 9, the results are shown as comparisons between the values from the first measurement described as day 1 and the results from the second measurement described as day 28 within the NaCl, DOX, APLN 40, and ELA 40 groups.

#### 3.2.1. Comparison of Ejection Fraction Between Day 1 and Day 28 in the Groups

One-way ANOVA followed with post hoc Duncan test showed no statistically significant differences between measurements taken on day 1 and 28 of the experiment in the Control group and the APLN 40 treatment. One-way ANOVA showed the significant effect of the DOX treatment on day 1 vs. day 28 (F = 1.12 = 29.95, *p* < 0.0001), * *p* < 0.0003 (post hoc Duncan test), and ELA 40 treatment (F = 1.14 = 7.84, *p* < 0.01), ** *p* < 0.01 (post hoc Duncan test) (Figure 6).

#### 3.2.2. Comparison of Fractional Shortening Between Day 1 and Day 28 in the Groups

One-way ANOVA followed with post hoc Duncan test showed no statistically significant differences between measurements taken on day 1 and 28 of the experiment in the Control group and the APLN 40 treatment. One-way ANOVA showed the significant effect of the DOX treatment on day 1 vs. day 28 (F = 1.12 = 31.34, *p* < 0.0001), * *p* < 0.0002 (post hoc Duncan test), and ELA 40 treatment (F = 1.14 = 8.14, *p* < 0.01), ** *p* < 0.01 (post hoc Duncan test) (Figure 7).

#### 3.2.3. Comparison of Stroke Volume Between Day 1 and Day 28 in the Groups

One-way ANOVA followed with post hoc Duncan test showed no statistically significant differences between measurements taken on day 1 and 28 of the experiment in the Control group, APLN 40, and ELA 40 treatment. One-way ANOVA showed the significant effect of the DOX treatment on day 1 vs. day 28 (F = 1.12 = 9.1, *p* < 0.01), * *p* < 0.01 (post hoc Duncan test) (Figure 8).

#### 3.2.4. Comparison of Cardiac Output Between Day 1 and Day 28 in the Groups

One-way ANOVA followed with post hoc Duncan test showed no statistically significant differences between measurements taken on day 1 and 28 of the experiment in the Control group, APLN 40, and ELA 40 treatment. One-way ANOVA showed the significant effect of the DOX treatment on day 1 vs. day 28 (F = 1.12 = 26.67, *p* < 0.0002), * *p* < 0.003 (post hoc Duncan test) (Figure 9).

## 4. Discussion

A growing body of evidence exists supporting the important role of the apelinergic system in modulating cardiovascular homeostasis in various diseases. Stimulation of this system results in vasodilation, improved angiogenesis, reduced inflammation, inhibition of pathological cardiac remodeling, and increased myocardial contractility [40,41]. Moreover, it affects the cell signaling mechanism via regulation of various ionic currents. Therefore, activation of the apelinergic system should be viewed as a possible strategy against chemotherapy-induced cardiotoxicity. To our knowledge, there are no preclinical studies determining the effect of the APLN in inhibiting changes in ECG parameters associated with DOX toxicity. Very few reports exist related to the influence of components of the apelinergic system on ECG parameters in different models of cardiotoxicity.

Our study showed that both APLN 40 and ELA 40 prevent DOX-induced prolongation of QT and QTc intervals. Moreover, APLN 40 and ELA 40 lead to a shortening of the QRS complex duration and an increase in HR, respectively. However, we observed contrary results in the APLN 200 and ELA 200 groups. In animals receiving higher doses of APJ agonists in combination with DOX, the prolongation of the QT and QTc intervals corresponds to the changes observed in the DOX group. Prolongation of QT and QTc intervals also occurred in the ML221 group. It seems that APLN and ELA affect cardiac electrical activity in a dose-dependent manner. Although lower doses of APJ agonists prevent changes induced by chronic DOX administration, higher doses of these drugs exert no cardioprotective effect. The observed outcome may be attributable to the toxicity of hyperstimulation of the apelin/elabela–APJ axis or clathrin-mediated APJ endocytosis though a β-arrestin-dependent mechanism [42]. The internalization of APJ has been shown to contribute to pathological cardiac remodeling via the p-S6K1, MEK/ERK, NO, MAPK, and PI3K/AKT signaling pathways [43]. It was discovered that the C-terminal Phe of the pyroglutamyl form of apelin-13 interacts with Phe^225^ and Trp^259^ of the APJ receptor, which is crucial for the internalization process [44]. Moreover, APJ internalization and desensitization exhibit a dose-dependent response [45]. A notable constraint of the present study is the absence of an apelin and elabela intermediate dose, between 40 and 200 µg/kg b.w./day. Consequently, further research is necessary to establish the effects of such a medium dose on ECG and TTE parameters.

Existing findings on apelinergic system effects on ECG parameters are in line with our research. Yassin et al. (2023) showed that APLN (15 ug/kg/day ip, for 2 weeks) shortened the QRS complex and corrected QT intervals compared to the control rats (female Wistar) [46]. In Li and co-workers’ research (2019) on a rat (male Sprague–Dawleys, age 2 months, weight 220–250 g) model of acute myocardial infarction (MI) which is involved in the arrhythmogenic mechanism and raising the risk to initiate Torsade de Pointes, pretreatment with APLN (15 μg/kg, ip) shortened the MI-induced QTc and QT interval [29]. This effect is probably related with the APLN ability to enhance the IK1/Kir2.1 channels’ currents through the IP3K pathway [29]. The duration of the QRS complex and QT segment on the ECG are closely related to the sodium current. Chen et al. (2012) proved that APLN significantly changed the atrial electrophysiology with a shortening of action potential duration, via regulation of various ionic currents [47]. According to Cai and the group (2023), APLN via APJ increases sodium-hydrogen activity, which leads to intracellular alkalinization, and an increased myofilament sensitivity for calcium. Thus, modulation of intracellular Na^+^ concentrations and turnover of sodium-calcium exchangers enhances intracellular calcium levels. This means that modulation of sodium current by APLN may play a major role in its inotropic effect [48]. Moreover, APJ can activate protein kinase C (PKC), an enzyme that is involved in the modulation of sodium current by APLN, since chelerythrine, the specific inhibitor of PKC, abolishes the increase in the sodium current by APLN [48].

Studies of the apelinergic system and ECG parameters are reflected also in several clinical trials. For example, increased serum ELA levels during MI were associated with higher HR and a higher incidence of atrioventricular conduction disturbances at the 12-month follow-up. Furthermore, reduced APLN levels during MI negatively correlated with Q/QRS [49]. In another study, in children with heart failure due to congenital heart disease, serum APLN levels showed a negative correlation with HR, and low APLN levels were associated with an unfavorable prognosis in heart failure [50].

Cancer therapeutics-related cardiac dysfunction (CTRCD) was repeatedly confirmed in preclinical and clinical studies [51,52,53]. However, TTE became an important diagnostic tool used in animal cardiotoxicity models, clinical studies, and in monitoring of patients during cancer therapy [51,54,55]. According to the recommendations of the American Society of Echocardiography (ASE) and the European Association of Cardiovascular Imaging (EACVI), one of the main echocardiographic criteria that allows us to confirm anthracycline-induced cardiotoxicity is assessment of the left ventricular ejection fraction (LVEF), the parameter determining of LV systolic function [51]. TTE performed in our study showed a decrease in LV contractility in the DOX group (lowered LVEF and FS and as a consequence a decrease in SV and CO on day 28 of the experiment) (Figure 6 and Figure 7). These changes are typical for DOX-induced cardiomyopathy [51,55]. However, a significant increase was observed in LVEF, FS, SV, and CO on day 28 of the experiment (improvement in LV systolic function) in the APLN 40 and ELA 40 groups (echocardiographic parameters obtained by us fit the norm for adult rats weighting 364 ± 44 g) (Figure 6, Figure 7, Figure 8 and Figure 9) [39]. Our results confirming positive inotropic actions of APJ agonists are in line with results of previous preclinical and clinical studies [56,57,58]. As suggested in our experimental model, combined therapy with DOX and APJ agonists to avoid DOX-induced conduction disturbances and heart failure supports current studies on development of effective anthracycline therapy free of cardiac complications. In particular, the echocardiographic findings are consistent with the results of our electrocardiographic analysis, supporting the cardioprotective properties of APJ stimulation. Dexrazoxane has been observed to induce similar effects on electrocardiographic and echocardiographic parameters. In 8-week-old DOX-treated Wistar rats, dexrazoxane administration prevented impairment of left ventricular systolic function, characterized as preserved LVEF and FS [59]. In patients diagnosed with aggressive non-Hodgkin’s lymphoma, dexrazoxane supplementation led to a substantial reduction in QT and QTc dispersion [60]. Furthermore, in a long-term observational study of childhood cancer survivors, dexrazoxane administration was associated with higher FS and LVEF nearly 20 years after primary exposure to anthracyclines [61]. In contrast, statin supplementation during anthracycline chemotherapy does not demonstrate the cardioprotective properties observed in ECG and TTE. In C57BL/6 mice treated with doxorubicin, the reduction in FS and EF was not ameliorated by lowastatin [62,63]. However, lowastatin appears to alleviate DOX-induced diastolic dysfunction, characterized by E-wave acceleration time [63]. Moreover, a double-blind, placebo-controlled clinical trial revealed that atorvastatin coadministration in the absence of existing indications had no effect on left ventricular systolic function [64]. Consequently, the stimulation of the apelinergic system may present an alternative to dexrazoxane, with outcomes that could exceed those of statins.

## 5. Conclusions

To our knowledge, we are the first to report a comprehensive evaluation of the influence of ELA on electrocardiographic changes in an in vivo preclinical model. Furthermore, we have shown that APJ stimulation counteracts DOX-induced QT prolongation. Our findings provide mechanistic support for the use of APLN, ELA, and their analogues as potential cardioprotective agents against anthracycline-induced cardiotoxicity. However, further research is necessary to investigate the molecular mechanisms of action, pharmacokinetics, and drug safety profile.

It seems particularly important to conduct preclinical studies in models of DOX-induced heart failure combining electrophysiological methods with electrocardiographic and echocardiographic diagnostics to expand the knowledge of these mechanisms.

## Data Availability

The data presented in this study are available on request from the corresponding author due to legal or ethical reasons.
